# Children in the 2015 South Indian floods: community members’ views

**DOI:** 10.1080/20008198.2018.1486122

**Published:** 2018-06-26

**Authors:** Revathi N. Krishna, Kevin R. Ronan, Eva Alisic

**Affiliations:** a Monash University Accident Research Centre, Monash University, Melbourne, Australia; b School of Human, Health and Social Sciences, Central Queensland University, Rockhampton, Australia; c Trauma Recovery Lab, Monash University Accident Research Centre, Monash University, Melbourne, Australia; d Child Health and Wellbeing Program, Melbourne School of Population and Global Health, University of Melbourne, Melbourne, Australia

**Keywords:** child-centred disaster risk reduction, traumatic stress, vulnerability, low- and middle-income countries, youth, disaster recovery, disaster risk reduction, disaster resilience education, CC-DRR, estrés traumático, vulnerabilidad, LMIC, juventud, recuperación a desastre, reducción del riesgo a desastre, educación de resiliencia ante desastres, CC-DRR, 创伤性应激, 易感性, LMIC, 青年, 灾难恢复, 减少灾害风险, 灾难韧性教育, • Disasters have negative impacts on children with gender caste and socioeconomic status playing an important role in the safety and recovery of the children and families from the floods. • Parents felt helpless and were dismayed by their inability to provide children with basic necessities during the floods.• Children worried about recurrence of floods and suffered from nightmares and from anxiety especially when it rained.• Community members’ suggestions are to involve children and themselves in the development and implementation of disaster resilience education programmes about flood awareness, preparedness, etc.

## Abstract

Little is known about children’s experiences and involvement in disaster preparation and recovery, in particular in low- and middle-income countries. Eliciting community members’ perspectives on the 2015 floods in Tamil Nadu, India, may generate useful insights for improving services in low-resource settings. This qualitative study aimed to understand how children in Chennai experienced the floods, as reported by the adults in their community, and to explore children’s involvement in disaster preparedness, response and recovery efforts as reported from the adults’ perspective. We conducted in-depth, semi-structured interviews (*N* = 48) with family members (*n* = 36), and with staff of non-governmental organizations (NGOs) (*n* = 12) who actively participated in relief and recovery efforts. We also conducted two focus group discussions (*n* = 14) with NGO staff about a year after the 2015 South Indian floods in Chennai, India. Six broad themes regarding children’s experiences and behaviours during and after the floods emerged: (1) unexpectedness of the floods; (2) children’s safety – barriers and facilitators; (3) parents’ reactions – helplessness, fear and pride; (4) children’s reactions – helping hands, fun and fear; (5) barriers to a return to ‘normal’; and (6) a determination to be prepared for next time. Children and families were deeply impacted by the floods, in part owing to a lack of preparation, as perceived by the study participants. It was also clear from the data analysis that caste and socioeconomic status played an important role in the families’ ability to evacuate safely. Helplessness on the part of the parents was apparent, as was children’s concern over recurrence of the flood. Similarly, gender appeared to affect child safety, recovery and other outcomes such as continued education. Priorities for future efforts involve the development and evaluation of child-centred education about flood awareness, child participation and safety.

## Abstract (es)

**Antecedentes**: Se conoce poco acerca de las experiencias de los niños y su participación en la preparación y recuperación de desastres, en particular en países de bajos y medios ingresos. La obtención de las perspectivas de los miembros de la comunidad sobre las inundaciones de 2015 en Tamil Nadu, India, puede generar reflexiones útiles para mejorar los servicios en entornos de bajos recursos.

**Objetivo**: Este estudio cualitativo apuntó a comprender a) Cómo experimentaron las inundaciones los niños en Chennai, según el reporte de los adultos en su comunidad, y b) explorar la participación de los niños en la preparación para el desastre, la respuesta y las labores de reconstrucción, según el reporte desde la perspectiva de los adultos.

**Método**: Realizamos entrevistas semi estructuradas en profundidad (N=48) con miembros de la familia (*n*=36), y con miembros de organizaciones no gubernamentales (*n*=12) que participaron activamente en las labores de ayuda y reconstrucción. También realizamos dos grupos de discusión (*n*=14) con miembros de ONG, aproximadamente un año después de las inundaciones del sur de la India en 2015 en Chennai, India.

**Resultados**: Surgieron seis amplios temas respecto a las experiencias de los niños y las conductas durante y después de las inundaciones: a) Lo inesperado de las inundaciones; b) Las barreras y facilitadores para la seguridad de los niños; c) La reacciones de los padres - impotencia, miedo y orgullo; d) Las reacciones de los niños – ayuda, diversión y miedo; e) Las barreras para el retorno a lo ‘normal’, y f) Una determinación para estar preparados para una próxima vez.

**Conclusión**: Los niños y las familias estuvieron profundamente impactados por las inundaciones, en parte debido a una falta de preparación, según la percepción de los participantes del estudio. También fue claro desde el análisis de los datos que el status socioeconómico y de castas jugó un rol importante en la capacidad de las familias para evacuar en forma segura. La impotencia de parte de los padres fue evidente, así como también la preocupación de los niños por la recurrencia de la inundación. Así también, el género pareció afectar la seguridad, recuperación y otros resultados de los niños, como la continuidad en la educación, etc. Las prioridades para los esfuerzos futuros incluyen el desarrollo y evaluación de la educación centrada en los niños acerca de la concientización de las inundaciones, participación de los niños, y seguridad.

## Abstract (zh)

背景：我们对于儿童在灾难准备和恢复的体验和参与度了解甚少，特别是在低收入和中等收入国家中。 社区成员对印度泰米尔纳德邦2015年洪灾的看法可能会为改善低资源环境下的服务产生有益的见解。

目标：这项质性研究旨在了解a）钦奈的儿童在洪水中的经历，是否正如其社区成年人所报告的那样; b）从成年人的角度来看，探索儿童参与防灾，应对和恢复的努力。

方法：我们与家庭成员（*n* = 36）和积极参与救灾和恢复工作的非政府组织的工作人员（*n* = 12）进行了深入的半结构化的访谈（N = 48），我们还在2015年南印度洪水后约一年时间内在印度金奈与非政府组织工作人员进行了两次焦点小组讨论（*n* = 14）。

结果：讨论得出了六个关于儿童在洪水期间和之后的经历和行为的广泛主题：a）洪水的意外发生; b）儿童的安全 - 障碍和促进因素; c）父母的反应 - 无助，恐惧和自豪; d）儿童的反应 - 援助，乐趣和恐惧; e）恢复到‘正常’的障碍，f）决定下次做好准备。

结论：研究参与者认为，儿童和家庭深受洪灾影响，部分原因是缺乏准备。 数据分析显示，种姓和社会经济地位对家庭安全撤离的能力起着重要作用。 父母的无助表现，以及儿童对洪水会重新出现的担心都很明显。 同样，性别似乎影响到儿童的安全，康复以及其它结果（如继续教育等）。未来工作的优先考虑应以包括制定和评估以儿童为中心防洪意识教育，儿童参与和安全。

## 1. Introduction

Exposure to potentially traumatic events in one’s lifetime is common. Age, female gender, low socioeconomic status, loss of income, racial/ethnic minority and education are key predictors of resilience (Bonanno, Galea, Bucciarelli, & Vlahov, 2007), in addition to the more commonly known elements such as a person’s mental health history, severity of trauma, length of exposure and own appraisal of the event.

Children represent the largest population segment in low- and middle-income countries (LMICs) and are often the first and most badly affected victims in natural disasters (Martin, 2010; Norris, Baker, Murphy, & Kaniasty, 2005). In addition to being exposed to physical injuries and potentially traumatic events (Mitchell & Borchard, 2014; Norris et al., 2005), children can become separated from caregivers and therefore are vulnerable to exploitation or abuse (Taylor, 2014). They may be confronted with a lack of food, shelter and social support (Babugura, 2008), and an inability to make sense of their surroundings, leading to a decreased ability to cope and increased vulnerability (UNICEF, 2006). Furthermore, disasters challenge all levels of the socioecological system in which the children are embedded, making it hard for children to be able to make sense and cope with the event (Masten, 2014; Masten & Narayan, 2012). Long-term mental health problems related to natural disasters have been extensively documented (e.g. Dogan, 2011; McDermott & Cobham, 2014). Thus, it is important to prepare children for such events as well as increase their resilience. Any intervention that aims to foster resilience needs to have a multisystemic approach (Masten, 2014) in order to be effective, accounting for the complexity of children’s environment, their experiences and other factors across various systems: biological, micro, meso, exo, macro and chrono (Ungar, Ghazinour, & Richter, 2013). A systematic review found that interventions that were culturally and contextually adapted resulted in creating a more positive impact on the recipients and their communities (Jordans, Pigott, & Tol, 2016).

The United Nations Sendai Framework has identified children and youth as agents of change and advocated for their active involvement in preparedness activities (UNISDR, 2015). Children can play an active and valuable role in the development and application of strategies and practices to minimize disaster risks and vulnerabilities (Amri, Haynes, Bird, & Ronan, 2017; Ronan et al., 2016). Although there is preliminary support for this stance (e.g. Amri et al., 2017; Ronan et al., 2016), it has not yet translated into larger scale, action-oriented, active involvement of children, worldwide, including in India (e.g. Joerin, Steinberger, Krishnamurthy, & Scolobig, 2017). To understand vulnerabilities and opportunities for active involvement of children in disaster preparedness and risk reduction, and for better support of their post-disaster mental health and well-being in LMICs, better insight into their psychosocial circumstances during and after disasters is needed.

### 1.1. Aims of the study

The current study aimed to generate insight into families’ experiences of being affected by the 2015 floods in Tamil Nadu, India, with a specific focus on the circumstances of children in communities that experience poverty. Secondly, we explore children’s involvement in disaster preparedness, response and recovery efforts as reported from the adults’ perspective. This study brings together the perspective of flood-affected community members and staff of non-governmental organizations (NGOs) who contributed to relief efforts after the floods. It is the starting point for a larger project on child-centred disaster risk reduction in Tamil Nadu.

### 1.2. Context: Tamil Nadu and the 2015 Chennai floods

Worldwide, India is one of the most disaster-prone countries owing to its geoclimatic conditions, high degree of socioeconomic vulnerability and population size. Tamil Nadu has the second longest coastline in India, which was significantly impacted by the 2004 Indian Ocean tsunami, causing 7793 direct deaths in the state. About 52% of Tamil Nadu’s 72 million population live in rural areas (Indian Census, 2011), with an estimated 12 million people living on or below the poverty line. Moreover, problems with class, caste, gender, and inter-district and urban–rural disparities are common (Harriss, Jeyaranjan, & Nagaraj, 2010; Vithayathil & Singh, 2012). Children aged between 0 and 14 years make up almost a quarter of the total population (National Family Health Survey-4, 2015–2016).

The 2015 floods in Tamil Nadu were caused by heavy rainfall during the north-west monsoon season in November/December 2015. Over 200 people were killed and over 1.8 million people were displaced (The International Federation of Red Cross and Red Crescent Societies; IFRC, 2015). With estimates of damage and losses of over 15 billion US dollars, the floods were one of the most expensive disasters of 2015 globally (EM-DAT, 2016). Heavy rains and flooding washed away roads and severed rail links. According to the Tamil Nadu government, about 3 million families of low socioeconomic communities suffered total or partial damage to their houses (Parliamentary Standing Committee on Home Affairs, 2016).

## 2. Methods

This study was conducted in urban and rural flood-affected communities living in poverty, in Tamil Nadu, India. The first author (RNK) collaborated with three NGOs working in the field of mental health, building on an already established relationship with the flood-affected communities. We obtained ethics approval from Monash University Human Research Ethics Committee. All ethics and data collection documentation was shared with the NGOs for their review. We encouraged the organizations to ask us any questions that they may have, and encouraged their staff to play an active role by not only introducing RNK to the communities, or being participants, but also reflecting on the interview guide and suggesting changes. In addition, one of the NGOs had an internal ethics committee that assessed all materials before the study started.

### 2.1. Participants and data collection

We used purposive sampling to capture experiences of a diverse group of people in these communities. Participants included affected family members and staff of three NGOs who worked on providing relief during the floods. We conducted semi-structured, in-depth interviews with families and staff participating in the study between December 2016 and February 2017, a year after the floods. During recruitment, we provided comprehensive information about the study to participants. However, owing to high illiteracy rates in the study communities, we recorded consent in audio format instead of in writing.

Topic guides for the interviews (Supplementary files A and B) included key themes such as family members’ and children’s experience during the floods, relevant support systems and their thoughts on future disaster preparedness. Although the topic guides were developed in advance, the guide was flexible and modified as themes developed or depending on the context, as required. For example, in December 2016, the study areas were also impacted by Cyclone Vardah. Therefore, we asked participants about insights they gained from the floods and how they used them to prepare to mitigate the effects of the cyclone. To ensure adequate distinction between the two incidents, we used interview strategies such as clarifying timelines and follow-up questions. In addition to the interviews, we conducted two focus group discussions (topic guide, Supplementary file C), with staff focusing on their observations of the communities during floods and future research directions. The interviews were conducted in Tamil by RNK at the participants’ preferred venue, usually in their houses or empty communal areas. Demographic characteristics of the participants are presented in Table 1. The focus group discussions lasted between 90 and 140 minutes and individual interviews were between 30 and 90 minutes. All focus group discussions and interviews were audio-recorded.

**Table 1.** Demographic characteristics of individual, in-depth interview participants (*N* = 48).

Demographic characteristics	Staff of NGO (*n* = 12)	Community members (*n* = 36)
Age (years), mean, (range)	36.1 (26–55)	34.4 (19–67)
Gender, *n* (%)		
Male	3 (25)	14 (39)
Female	9 (75)	22 (61)
No. of children		
0^a^	4	7
1–2	6	19
≥ 3	2	10
No. of participants with at least one child **< **18 years	6	24
Age of children < 18 years (years), mean (range)	8.67 (3–17)	7.43 (0.5–17)

^a ^All participants were either living with a child in a joint-family set-up or working closely with children, whether or not they had children younger than 18 years of their own.

NGO, non-governmental organization.

### 2.2. Analysis

All interviews and focus group discussions were translated and transcribed into English from Tamil. RNK read and reread the transcripts to gain familiarity with raw data. Since this study is part of a larger project, we used the data exclusively about children. Analysis was inspired by Corbin and Strauss’s (1990) grounded theory, using a constant comparison approach. Consistent with the grounded theory approach, we started to analyse the data as data collection progressed by creating a list of overarching themes which continued to be updated throughout the data collection and analysis process. This was aided by the use of interview notes and a debriefing form after each interview, which also included potential changes that needed to be made to the topic guide, observation of main themes and barriers to interviews. Next, the transcripts were uploaded into and analysed using NVivo version 11 (QSR International Pty Ltd, 2015) qualitative data analysis software. First, we started the process of open coding, by identifying new codes that emerged from the data. We then compared these new codes with some of the themes/codes that we expected to see a priori and merged them with the list of themes we had developed. All the coding and analysis of data were completed in NVivo. Two authors (RNK and EA) continuously discussed the coding strategy by looking at the coding summary report within NVivo for each node and the codebook until these were finalized. To avoid any data overlap with the other study, we used ‘CH’ (children) at the beginning of every node that was relevant and used for this study. During the coding process, we continued to develop higher and lower order codes and started to link them with one another as well, to prevent repetition within the codes as well as not miss relevant data. Once we had completed the coding process for all interviews and focus group discussions, we sorted the data according to their appropriate themes and sub-themes to look for any errors in coding and recoded the data where necessary. KRR examined the final themes and codes, and their supporting data (quotes) and relations, with discrepancies discussed and resolved among the authors.

## 3. Results

**Figure 1.**
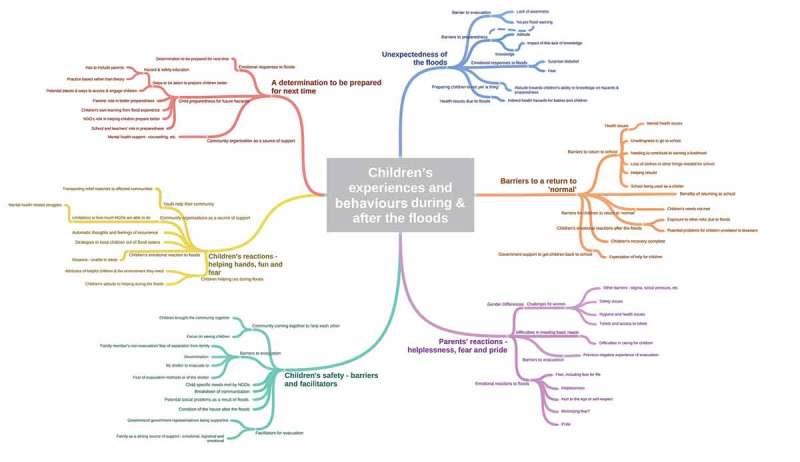
Children’s experiences and behaviours during and after the floods as observed by community members.

Six broad themes and a number of subthemes emerged regarding children’s experiences and behaviours during and after the floods (Figure 1): (1) unexpectedness of the floods; (2) children’s safety – barriers and facilitators; (3) parents’ reactions – helplessness, fear and pride; (4) children’s reactions – helping hands, fun and fear; (5) barriers to a return to ‘normal’; and (6) a determination to be prepared for next time.

### 3.1. Unexpectedness of the floods

Most participants, community members and staff of NGOs alike, reported that the floods were a surprise; they happened unexpectedly and overnight, giving them no time to react. They reported that, although they are accustomed to ankle-deep water on their streets during monsoon, they had not expected water to enter their houses and to remain stagnant for days, despite the heavy rain for several days prior to the flooding.

My mother and we were shocked that the water came inside our house. At 4:30am when we woke up there was water everywhere – my mother and everyone started exclaiming, ‘there is water in our house, there is water in our house’ like she was in disbelief. (22-year-old male in an urban community)

Consequently, not only was the community unprepared for the floods, but the floods also had a distinct impact on the children. They reported that preparedness for hazards was not common practice in the community. Attitude towards and knowledge about hazards were mentioned as barriers, especially by staff of NGOs. Specifically, many participants, especially community members, reported that they did not know how to prepare for floods, while some others thought that it was beyond their control and nothing could actually prepare them for it.

My daughter said ‘papa how will the water reduce? There is still lots of water on the streets’. But I told her ‘nothing will happen, don’t be afraid. The water will reduce. If it does not and if our time has come, we all have to die one day anyway. So, don’t worry as everybody’s life is still at stake. But we gave her courage and hope.’ That’s all I could tell her, nothing else. (55-year-old male from a rural community)

In fact, many staff of NGOs reported that they only received training about hazards and how to help their communities or prepare for such events after the floods. However, the staff also reported that they did not receive any particular training specific to helping children to prepare.

### 3.2. Children’s safety – barriers and facilitators

Regardless of the community’s preparedness, most participants reported that the community came together to ensure the children’s safety. Community members as well as staff of NGOs reported and provided examples of times when children brought some of the families in the communities together despite their previously strained relationships:

Let’s say two neighbours don’t talk to each other. They sent their kids to find out how the other family was doing and if they needed anything. If they needed something, then, the families sent it via the kids. So, they didn’t necessarily talk to the other family, but, helped them nonetheless using the children. Many people did this. (Focus Group Discussion with NGO staff)

Despite this, some families reported struggling to ensure their children’s safety owing to discrimination. The caste system in India, and the role it plays in the social hierarchy, meant that families were unable to move into evacuation shelters with other families in the village:

A few people were brought to the community hall and the others there enquired about those people and why they have been brought to the hall and said that they have been insulted because those people were brought in … Even when those people [from a lower caste] requested to be allowed into the hall, they [the higher caste people] did not allow them. (25-year-old male from a rural community)

Nonetheless, in some cases, village heads or representatives provided support to the communities irrespective of caste, which was a significant facilitator for evacuation. Staff of NGOs reported the caste system being a significant barrier not only to safe evacuation, but also to the process of recovery.

Initially, we were told that everyone was to stay in the hall. We do not belong to their community [caste] and so they were not accommodative of us. Later, the leader came and convinced them to share the hall with us. (25-year-old male from a rural community)

Those who could evacuate reported doing so by whatever means was available to them, whether it was walking or on boats; in children’s cases, often, it was on the shoulder of their parents or family. Families reported evacuating to community halls or schools. Some families who could send their children to the house of relatives or friends who were unaffected by floods reported doing so, although this often led to worries about family members’ safety owing to the interruption of telephone networks. In addition to discrimination, fear regarding the evacuation method (boats that were meant to be on the ocean were on roads, making sharp turns, etc.), fear of separation from family members and lack of an evacuation shelter were barriers to evacuation for children.

My family told me that if something were to happen, then, it would be best to be together and go away [indicating death] together. So, my children didn’t go to my in-law’s house. (40-year-old male in a rural community)

I saw many newborns, pregnant women, elderly all getting on the boat with much fear. In an ocean there is nothing to interrupt the boat, but, here, the boat kept hitting the walls, corners, steps, etc. and because of this, the boats would shake and sometimes even flip over, putting all the people on the boat in the water to be rescued. (46-year-old woman staff of an NGO)

Furthermore, waterborne diseases were mentioned as a threat, as homes were infested with snakes and insects after the water had receded. Finally, staff from NGOs reported that child safety and well-being were compromised by a cascading effect of secondary stressors, including discontinuation of education (which primarily affected girls owing to financial constraints), and child marriage as a way to protect the girl and to lessen the parents’ financial burden:

After a disaster, many times, the government goes around giving gifts [as financial support] – they give sarees and thalis [the sacred thread tied around a woman’s neck during a marriage ceremony] to the affected families. They never stop and ask if the child who is getting married is 18 years old, do they? So, yes, when the families have consented, these things happen without much thought. (48-year-old woman staff of an NGO)

### 3.3. Parents’ reactions – helplessness, fear and pride

Participants reported feeling helpless during the floods; they reported finding it incredibly hard to care for their children. Although many participants had been evacuated or had a dry place to stay, providing children with basic necessities such as food, water and safety was particularly difficult. Parents reported feeling despair and anguish as they and or their children had to ‘beg’ for food and water.

We were hurt because we couldn’t even feed our kids and were wondering which self-respecting person would stand in a queue to get food. (45-year-old woman staff of an NGO)

Parents reported generally being fearful for their own and their children’s safety, especially since the floods were not something that they had expected. Despite these negative feelings, parents described feeling proud when their children helped them and others.

My daughter didn’t get scared at all – she was as good as a boy, full of courage. Not only did she manage to be safe, she also brought me out of the water. (45-year-old woman in an urban community)

Conversely, many parents seemed to minimize the fear that children felt:

No, what’s there to fear? She wasn’t afraid. (55-year-old male in a rural community)

Gender differences stood out: women and girls were described as having a harder time in the shelters, with issues related to privacy, access to toilets and general safety. They were afraid to sleep, fearing kidnapping or sexual assault. Essentials such as clean clothes, menstrual products and privacy were not available. They were additionally challenged by societal gender roles and social stigma.

In camps, women and adolescent girls didn’t sleep at all because … they were scared of being sexually assaulted. They couldn’t also ask for sanitary products – they were too embarrassed … Men were everywhere, how would they go to the toilet, they couldn’t even change clothes or underwear because of lack of privacy. (46-year-old woman staff in an NGO)

### 3.4. Children’s reactions – helping hands, fun and fear

Participants reported that many children thought it was fun to play in the water. They enjoyed the rain and wanted to play in the water, and were not as scared as their parents.

Children were free, there was no problem. They were happy to play in the water. If they got whatever they needed (e.g. biscuits), they were happy. (52-year-old woman in an urban community)

In addition to being bridges between families, as reported in Section 3.2, children were reportedly eager to participate in the relief efforts. Participants identified characteristics such as being smart, active and brave, and having parents who helped, as influencing children’s altruistic attitude. Participants reported that children (as young as 10–12 years old) contributed to the relief efforts by helping the youth to push three wheeled bikes to distribute relief material even when they were asked to stay home.

The children, those who were over 12 years old (there are only three of them in our community) got into the water and helped the adults in moving and pulling boats and also made arrangements for food. (22-year-old male in an urban community)

When the water levels rose, children tended to be afraid. Many parents reported that their children had a difficult time after the floods when they lost their books, toys, pets and other things they might have held dear prior to the floods:

Adults only worried about their families, but, children tended to worry about their books, things, laptops, certificates, etc. So, children really had a hard time. As adults, we tend to understand, but, children cannot understand and so, it was much harder for the children in general. (54-year-old woman from an urban community)

Parents recognized that keeping children out of flood waters was a hard task for them and had to resort to punishing them in order to keep them out of the water.

I advised my child not to play in the water. But, he wouldn’t listen to me – he’d come to the water and play. Since we have all the fear about the water as it would drown us, we try to beat the children to ensure safety. Yet children do not listen and they are only interested in playing in the water. (23-year-old woman from a rural community)

Many parents discussed how hard it must have been for children to see their parents in distress and their houses in disarray, needing to leave the house at a moment’s notice and not knowing in what state they would find their homes upon return.

They [the children] were scared that we might have lost all our things back home because none of us knew what would have happened. We only saw water [everywhere]. Not only were my younger kids worried, my older kids were also very worried. (45-year-old woman from a rural community)

A number of parents reported that children were scared and often found themselves experiencing nightmares about the floods and having trouble sleeping. In fact, many parents reported that every time it had rained since the floods, their children worried that their community might be flooded again.

As they started seeing these kinds of things and even experiencing it themselves, they have developed a fear. This fear hasn’t even gone away till today – if it rains, or there is strong winds, they ask me if it’s going to be a problem again. The fear is still with them because of how much they were affected by it [floods] personally. (36-year-old male staff of an NGO)

### 3.5. Barriers to a return to ‘normal’

Thoughts of recurrence were not the only barrier for children’s return to normality. Children’s ill-health (e.g. skin diseases, coughs, colds and fever) and loss of books, uniforms, etc., were common reasons for children’s inability to return to school during or right after the floods. Other reasons included schools being used as a shelter, children needing to contribute to the family’s livelihood and helping to rebuild their house, and children’s unwillingness to go to school.

I think it took them [children] about two or three months to get back to school. Though the attendance improved after giving them some books and uniforms, it still took them a good few months to get back to school. (45-year-old woman from an urban community)

Parents reported that when children returned to school, exams were nearing and children were often anxious because they were expected to perform well despite having lost their books. Staff of NGOs reported that they met with teachers to help the teachers deal with their own traumatic experience with the floods and be more understanding towards children and their situations.

Teachers couldn’t teach, if they taught, they would be strict – just like before. But, that is not possible right? They were mad at the students, and started to put pressure on the kids to study because of the half-yearly exams that were coming up. They wanted to complete the assigned syllabi before the exams and couldn’t. (46-year-old woman staff of an NGO)

The staff of NGOs also thought that every agency, including the government, focused primarily on relief aid distribution and neglected mental health and well-being. They reported that their attempts to meet children’s needs were insufficient.

Everyone focused on relief material, but, not on mental health. I think schools should have counselling for children and that must have been a priority. Not just for the students, but for the teachers too. (46-year-old woman staff of an NGO)

On the other hand, children continued to be anxious about the recurrence of floods. This was especially clear when we asked them about the effects of and preparation for Cyclone Vardah, which had recently affected the communities. The children reportedly made their parents promise them that they would come and get them if it started to rain heavily. Parents reported that children took a couple of months following the floods to resume their usual routines. Many felt anxious after the floods, refusing to move back to their homes. Parents identified benefits to children returning to school; it helped children settle into their routines more quickly.

Everything settled down after they went back to school and slowly they started getting over their fear. (52-year-old woman from an urban community)

As mentioned earlier, children were exposed to secondary stressors such as increased exposure to domestic violence, parental alcoholism, potential abuse and other problems that tend to emerge or be exacerbated during crises. Children’s needs were reported to be largely unmet, even in the recovery phase. However, these were primarily identified and reported by staff of NGOs and not much by community members.

Even in camps, girls suffered a lot of abuse. There are a lot of children who said they did not get food, water, clothes, or anything, but, their father was able to get alcohol. That [alcohol] was available during the flood, but, children’s basic necessities were not met. (Focus Group Discussion with NGO Staff)

### 3.6. A determination to be prepared for next time

Parents and staff reported their determination to be prepared for future disasters.

I have made some resolutions that if this were to happen again, I will be better prepared. (28-year-old woman from a rural community)

The community members suggested many ways to be better prepared for future floods. Participants reported that it would be beneficial for children to learn about disasters and emergency procedures as part of their school curricula. This included ideas about the form of education, i.e. children would benefit if they were practice based rather than lecture based. NGO staff emphasized that both children and parents should be educated in emergency procedures to minimize confusion. They also recommended using engaging methods such as street plays, theatre and songs to teach children and the community about disaster preparedness.

It’s really easy to reach children compared to adults. They tend to understand things faster, retain them in their memories longer. So, through theatre, fun and games, we can disseminate the information … Parents and children need to be taught these things – when they both learn, then, when they face an emergency situation, their knowledge will be comparable and won’t be doing different things. (Focus Group Discussion with NGO staff)

To reach children who do not attend school, participants suggested community-based activities and the use of tuition centres where children tend to gather for after-school activities.

Even though mental health was not something that the participants discussed in detail, they all agreed that it was important to ensure children’s well-being. Their recommendations are shown in Table 2. Community members primarily suggested reassuring children and teaching them to help others as strategies to ensure children’s well-being. NGO staff, however, recommended specific mental health-related strategies, including child-friendly dissemination of information (Table 2).

**Table 2.** Community members’ recommendations to ensure children’s well-being and increased preparedness for a disaster.

Steps to ensure children’s well-being and increased preparedness in a disaster	Relevant quotes by participants
Create awareness about the disaster in children in a child-friendly manner	‘We need to tell children in simple terms and not scare them. We should tell them that if it rains too heavily, then, we might be flooded or even get washed away – but, we need to tell them this in a kind and child-friendly manner. This would make it easy for them to understand if not, they will get scared and upset.’ (36-year-old male staff of an NGO)
Reassure children that things will be well	‘I will give my child all the confidence to overcome [the flood] and tell her not to be afraid and be with me. That is what I could do.’ (23-year-old woman from a rural community)
Pack toys and things that children are attached to ahead of time	‘For example: her toys, or dolls or things like that. Even making sure that she had friends around – or kids of her age. It makes kids calm down better if they have things they like or friends.’ (Focus Group Discussion with NGO staff)
Involve children in preparing for floods and use their disaster experience (e.g. floods)	‘Several kids are quite resourceful as they have lots of ideas after they experienced these events recently. We can learn from them! They come forward and tell us!’ (38-year-old woman staff of an NGO)
Help children pack their books and things they need	‘Similar to the adults’ things, whatever children need, needs to be kept safely, needs to be protected and added to that bag – for example: their certificates, their electronic things, etc. Parents can help children pack things and help think through things they may need.’ (54-year-old woman from an urban community)
Instil a sense of generosity and altruism in children	‘I will teach them when the time is right – they are still very small. Will teach them how to rescue people by swimming with the current – not against it – and pulling up people by their hair/head – not trying to lift or carry them.’ (34-year-old male from a rural community)
Schools should provide mental health support – counselling for children affected by disasters	‘It is extremely important because children’s mental health is very important. These kids can only come up in life if they have the chance to education and being able to work hard. If they don’t get their education on time, food and nutrition on time, then, it makes it hard for the kids to be well and bounce back. We and schools should help children by giving them counselling.’ (Focus Group Discussion with NGO staff)

NGO, non-governmental organization.

## 4. Discussion

The present study gave voice to communities affected by the 2015 Chennai floods, with specific focus on children. Although it had been raining for days before the floods, participants were surprised by the flooding of their communities. Class and caste system emerged as barriers to safety and access to relief material; however, children were instrumental in overcoming some communication barriers within communities. Similarly, gender issues stood out as shaping recovery experiences. While community members identified some mental health symptoms, they did not make explicit connections to mental health. Recommendations for future preparedness and mental well-being were mostly offered by NGO staff. In this discussion, we reflect on these key social and mental health findings.

The caste system is rooted in religion and has been linked with socioeconomic inequality, with worse outcomes for women and children (Jungari & Chauhan, 2017). Caste system, gender and socioeconomic status have a significant influence on health, life expectancy, and other important although less conventional health determinants such as urbanization, poor access to water and sanitation, food insecurity, environmental degradation, social stratification and income inequality (Patel et al., 2015). The caste system emerged as a factor that influenced children’s access to relief and support after the floods. In India, people from lower castes have been identified as receiving less aid and having worse outcomes after disasters (Aldrich, 2010; Kumaran & Negi, 2006). Globally, social structure – race, ethnicity, caste and class – has been found to play a role in the ability to cope with and recover from disasters (Bolin, 2007), including receiving aid. For example, race was critical in the distribution of aid after Hurricane Katrina (Finch, Emrich, & Cutter, 2010; Fothergill, Maestas, & Darlington, 1999; Fothergill & Peek, 2004). We found that many families belonging to a lower caste and class could not move to a safer place because of the unwillingness of the higher caste people to share the shelter. These issues of discrimination based on caste and class go hand in hand with poverty, which is known to have a negative cyclical relationship with mental health (Patel & Kleinman, 2003) and building resilience (Masten, 2014).

This study also highlights the role of children and youth in overcoming communication barriers by bringing families closer, despite previous conflicts. Disaster research has identified children as effective communicators of risk (Mitchell, Haynes, Hall, Choong, & Oven, 2008; Plan International, 2010). Children’s knowledge of their community and its needs can translate into enhancing the adaptive capacities needed to address disaster risk (Finnis, Johnston, Ronan, & White, 2010; Haynes & Tanner, 2015). This demonstrates that children’s active involvement can potentially increase a community’s overall disaster preparedness and reduce vulnerability.

When gender is added to the mix of risk factors, the outcome appears to be even worse: we found that women and girls were marginalized and their needs were neglected. Women from a lower caste or class have a harder time recovering from disasters (Ray-Bennett, 2009). In addition to discrimination and lack of access to jobs, relief materials and income replacement/generation schemes, they suffer from a lack of privacy in the shelters and access to toilets, and increased incidences of domestic violence and sexual assault (Enarson, Fothergill, & Peek, 2007). Our study highlights the need for relief materials and distributors to be sensitive to gender-specific needs and prevailing social norms. Importantly, attention needs to be paid to girls who might drop out of school or be married off because of financial constraints or other social issues. Education can serve as a protective factor against child marriage, which increases the risk of domestic violence, pregnancy, and childbirth at a young age and its related complications. Furthermore, education may provide the youth, especially girls, with the tools necessary to potentially break out of the vicious cycle of poverty and mental ill-health.

Mental health issues were not directly acknowledged in most of our interviews. Although most families identified symptoms akin to traumatic stress (e.g. nightmares, anxiety about recurrence, general fear), they did not identify them as related to mental ill-health or needing help beyond reassurance by family members. For example: participants did not report any mental health issues as such; however, they reported their children having nightmares about being trapped in water, or not wanting to return to school unless their parents promised to come and get them if it started raining, especially during Cyclone Vardah. Regardless of families being able to connect these symptoms to mental health, they valued their children’s well-being. In LMICs such as India, awareness about mental health is scarce and mental health problems are often stigmatized, while policies prioritizing mental health are largely absent (Khandelwal, Jhingan, Ramesh, Gupta, & Srivastava, 2004; Patel, 2007; Srivastava, Chatterjee, & Bhat, 2016). Consequently, there is an urgent need to make mental health a priority (Patel, 2007) and for more mental health research (Sharan et al., 2009), specifically traumatic stress research (Fodor et al., 2014; Schnyder, 2013), to be conducted in these settings. Interventions developed to suit this population would benefit from attuning to this attitude to mental health and design interventions adapted to this context. Cultural adaptation and keeping contextual factors at the heart of an intervention align with much of resilience research (Masten, 2014; Ungar et al., 2013), including helping children to build resilience in conflict and complex emergency settings (Jordans et al., 2016; Tol, Song, & Jordans, 2013).

This study is limited by the fact that children were not interviewed as part of this study. Although we aimed to interview an equal number of men and women, we found that 65% of our participants were women. This could be attributed to interviews being conducted during the day, a lower level of interest in participating among men and the interviewer being a woman. Finally, while this could provide a representative sample of the communities we spoke to, these findings cannot be generalized to other LMICs or even other groups within India.

This study has implications for both research and practice. A similar study to understand the children’s experiences from the perspective of the children themselves will probably yield important information and the opportunity to further triangulate our findings. Our study adds to the growing literature calling for more research on traumatic stress from settings such as India in order to understand the unique cross-cultural perspective and to tailor interventions to suit this population. Future work could also build evidence around people’s experiences and their attitude towards preparedness, and clarify their unique contexts in other settings in India. There is also a need to simultaneously build evidence towards developing and implementing key safety messages and behaviours, with children at the heart of this process. To facilitate key messaging, and children’s safety and involvement, researchers, NGOs and government need to work together with children and communities. For such an intervention to be successful, it needs to be tailored, tested and implemented within a community’s way of life.

## References

[CIT0001] Aldrich D. P. (2010). Separate and unequal: Post-tsunami aid distribution in Southern India. [Special issue on inequality and poverty: American and International Perspectives]. *Social Science Quarterly*, 91(5), 1369–11. 21125763 10.1111/j.1540-6237.2010.00736.x

[CIT0002] Amri A., Haynes K., Bird D. K., & Ronan K. (2017). Bridging the divide between studies on disaster risk reduction education and child-centred disaster risk reduction: A critical review. *Children’s Geographies*. doi:10.1080/14733285.2017.1358448

[CIT0003] Babugura A. A. (2008). Vulnerability of children and youth in drought disasters: A case study of Botswana. *Children, Youth and Environments*, 18(1), 126–157.

[CIT0004] Bolin B. (2007). Race, class, ethnicity, and disaster vulnerability In Rodriguez, H. E. Quarantellli and R. Dynes (eds.). *Handbook of disaster research* (pp. 113–129). New York, NY: Springer New York.

[CIT0005] Bonanno G. A., Galea S., Bucciarelli A., & Vlahov D. (2007). What predicts psychological resilience after disaster? The role of demographics, resources, and life stress. *Journal of Consulting and Clinical Psychology*, 75(5), 671–682. 17907849 10.1037/0022-006X.75.5.671

[CIT0006] Corbin J., & Strauss A. (1990). Grounded theory research: Procedures, canons and evaluative criteria. *Zeitschrift Für Soziologie*, 19(6), 418–427.

[CIT0007] Dogan A. (2011). Adolescents’ posttraumatic stress reactions and behavior problems following Marmara earthquake. *European Journal of Psychotraumatology*, 2(1), 5825. 10.3402/ejpt.v2i0.5825 PMC3402128 22893811

[CIT0008] EM-DAT (2016). The OFDA/CRED international disaster database. Retrieved 716, 2016, from http://www.emdat.be

[CIT0009] Enarson E., Fothergill A., & Peek L. (2007). *Gender and disaster: Foundations and directions handbook of disaster research* (pp. 130–146). New York, NY: Springer.

[CIT0010] Finch C., Emrich C. T., & Cutter S. L. (2010). Disaster disparities and differential recovery in New Orleans. *Population and Environment*, 31(4), 179–202.

[CIT0011] Finnis K. K., Johnston D. M., Ronan K. R., & White J. D. (2010). Hazard perceptions and preparedness of Taranaki youth. *Disaster Prevention and Management: an International Journal*, 19(2), 175–184.

[CIT0012] Fodor K. E., Unterhitzenberger J., Chou C. Y., Kartal D., Leistner S., Milosavljevic M., … Alisic E. (2014). Is traumatic stress research global? A bibliometric analysis. *European Journal of Psychotraumatology*, 5(1), 23269. 10.3402/ejpt.v5.23269 PMC3930940 24563730

[CIT0013] Fothergill A., Maestas E. G. M., & Darlington J. D. (1999). Race, ethnicity and disasters in the USA: A review of the literature. *Disasters*, 23(2), 156–173. 10379098 10.1111/1467-7717.00111

[CIT0014] Fothergill A., & Peek L. A. (2004). Poverty and disasters in the United States: A review of recent sociological findings. *Natural Hazards*, 32, 89–110.

[CIT0015] Harriss J., Jeyaranjan J., & Nagaraj K. (2010). Land, labour and caste politics in rural Tamil Nadu in the 20th century: Iruvelpattu (1916-2008). *Economic and Political Weekly*, *45*(31), 47–61.

[CIT0016] Haynes K., & Tanner T. M. (2015). Empowering young people and strengthening resilience: Youth centred participatory video as a tool for climate change adaptation and disaster risk reduction. *Children’s Geographies*, 13(3), 357–371.

[CIT0017] The Indian Census (2011). Tamil Nadu population census data 2011. Retrieved from http://www.census2011.co.in/states.php

[CIT0018] International Institute for Population Sciences (2015–2016). *National family health survey, NFHS-4; State fact sheet: Tamil Nadu*. New Delhi: Ministry of Health and Family Welfare Retrieved from http://rchiips.org/NFHS/pdf/NFHS4/TN_FactSheet.pdf

[CIT0019] Joerin J., Steinberger F., Krishnamurthy R. R., & Scolobig A. (2017, 11). *Disaster recovery processes: Analysing the interplay between communities and authorities in Chennai, India* . Proceedings of 7th International Conference on Building Resilience: Using scientific knowledge to inform policy and practice in disaster risk reduction, Bangkok, Thailand.

[CIT0020] Jordans M. J., Pigott H., & Tol W. A. (2016). Interventions for children affected by armed conflict: A systematic review of mental health and psychosocial support in low-and middle-income countries. *Current Psychiatry Reports*, 18(1), 9. 26769198 10.1007/s11920-015-0648-z PMC4713453

[CIT0021] Jungari S., & Chauhan B. G. (2017). Caste, wealth and regional inequalities in health status of women and children in India. *Contemporary Voice of Dalit*, 9(1), 87–100.

[CIT0022] Khandelwal S. K., Jhingan H. P., Ramesh S., Gupta R. K., & Srivastava V. K. (2004). India mental health country profile. *International Review of Psychiatry*, 16(1–2), 126–141. 15276945 10.1080/09540260310001635177

[CIT0023] Kumaran T. V., & Negi E. (2006). Experiences of rural and urban communities in Tamil Nadu in the aftermath of the 2004 tsunami. *Built Environment*, 32(4), 375–386.

[CIT0024] Martin M. L. (2010). Child participation in disaster risk reduction: The case of flood-affected children in Bangladesh. *Third World Quarterly*, 31(8), 1357–1375. 21506299 10.1080/01436597.2010.541086

[CIT0025] Masten A. S. (2014). Global perspectives on resilience in children and youth. *Child Development*, 85(1), 6–20. 24341286 10.1111/cdev.12205

[CIT0026] Masten A. S., & Narayan A. J. (2012). Child development in the context of disaster, war, and terrorism: Pathways of risk and resilience. *Annual Review of Psychology*, 63, 227–257. 10.1146/annurev-psych-120710-100356 PMC5858878 21943168

[CIT0027] McDermott B. M., & Cobham V. E. (2014). A stepped-care model of post-disaster child and adolescent mental health service provision. *European Journal of Psychotraumatology*, 5(1), 24294. 10.3402/ejpt.v5.24294 PMC4095762 25045422

[CIT0028] Mitchell P., & Borchard C. (2014). Mainstreaming children’s vulnerabilities and capacities into community-based adaptation to enhance impact. *Climate and Development*, 6(4), 372–381.

[CIT0029] Mitchell T., Haynes K., Hall N., Choong W., & Oven K. (2008). The role of children and youth in communicating disaster risk. *Children, Youth and Environments*, 18(1), 254–279. http://www.jstor.org/stable/10.7721/chilyoutenvi.18.1.0254

[CIT0030] Norris F. H., Baker C. K., Murphy A. D., & Kaniasty K. (2005). Social support mobilization and deterioration after Mexico’s 1999 flood: Effects of context, gender, and time. *American Journal of Community Psychology*, 36(1–2), 15–28. 16134042 10.1007/s10464-005-6230-9

[CIT0031] North R. (2015, 12 1). Poorest hit hardest by south India floods. International Federation of Red Cross and Red Crescent Societies. Retrieved from http://www.ifrc.org/en/news-and-media/news-stories/asia-pacific/india/poorest-hit-hardest-by-south-india-floods-69690/

[CIT0032] Parliamentary Standing Committee on Home Affairs (2016). *Disaster in Chennai caused by torrential rainfall and consequent flooding* (198th report). New Delhi: Parliament of India, Rajya Sabha Retrieved from http://www.indiaenvironmentportal.org.in/files/file/Disaster%20in%20Chennai.pdf

[CIT0033] Patel V. (2007). Mental health in low- and middle-income countries. *British Medical Bulletin*, 81–82(1), 81–96. 10.1093/bmb/ldm010 17470476

[CIT0034] Patel V., & Kleinman A. (2003). Poverty and common mental disorders in developing countries. *Bulletin of the World Health Organization*, 81(8), 609–615. 14576893 PMC2572527

[CIT0035] Patel V., Parikh R., Nandraj S., Balasubramaniam P., Narayan K., Paul V. K., … Reddy K. S. (2015). Assuring health coverage for all in India. *The Lancet*, 386(10011), 2422–2435. 10.1016/S0140-6736(15)00955-1 26700532

[CIT0036] Plan (2010). *Child centered disaster risk reduction: Building resilience through participation*. London, UK: Author.

[CIT0037] Ray-Bennett N. S. (2009). Coping with multiple disasters and diminishing livelihood resources caste, class, and gender perspectives: The case from Orissa, India. *Regional Development Dialogue*, 30(1), 108–120.

[CIT0038] Ronan K. R., Haynes K., Towers B., Alisic E., Ireland N., Amri A., … Petal M. (2016). Child-centred disaster risk reduction: Can disaster resilience programs reduce risk and increase the resilience of children and households? *Australian Journal of Emergency Management*, 31(3), 49–58.

[CIT0039] Schnyder U. (2013). Trauma is a global issue. *European Journal of Psychotraumatology*, 4(1), 20419. 10.3402/ejpt.v4i0.20419 PMC3589434 23469313

[CIT0040] Sharan P., Gallo C., Gureje O., Lamberte E., Mari J. J., Mazzotti G., … Saxena S. (2009). Mental health research priorities in low-and middle-income countries of Africa, Asia, Latin America and the Caribbean. *The British Journal of Psychiatry*, 195(4), 354–363. 19794206 10.1192/bjp.bp.108.050187 PMC3432479

[CIT0041] Srivastava K., Chatterjee K., & Bhat P. S. (2016). Mental health awareness: The Indian scenario. *Industrial Psychiatry Journal*, 25(2), 131–134. 28659690 10.4103/ipj.ipj_45_17 PMC5479084

[CIT0042] Taylor G. (2014). Current measures to address the social vulnerability of children in disaster risk reduction-exploring the European Union’s disaster risk reduction strategy. *Planet@Risk*, 2(2), 77–84.

[CIT0043] Tol W. A., Song S., & Jordans M. J. (2013). Annual research review: Resilience and mental health in children and adolescents living in areas of armed conflict–A systematic review of findings in low‐and middle‐income countries. *Journal of Child Psychology and Psychiatry*, 54(4), 445–460. 23414226 10.1111/jcpp.12053

[CIT0044] Ungar M., Ghazinour M., & Richter J. (2013). Annual research review: What is resilience within the social ecology of human development? *Journal of Child Psychology and Psychiatry*, 54(4), 348–366. 23215898 10.1111/jcpp.12025

[CIT0045] UNICEF (2006). Child alert: Horn of Africa (A report on the impact of drought on children). New York: Author.

[CIT0046] UNISDR (United Nations International Strategy for Disaster Reduction) (2015). *Sendai framework for disaster risk reduction 2015–2030*. Geneva: UNISDR.

[CIT0047] Vithayathil T., & Singh G. (2012). Spaces of discrimination. *Economic & Political Weekly*, 47(37), 60–66.

