# Supplementary material for: Children in the 2015 South Indian floods: community members’ views
Source: Eur J Psychotraumatol. 2018 Jun 26;9(Suppl 2):1486122. doi: 10.1080/20008198.2018.1486122 (PMC6038026; doi:10.1080/20008198.2018.1486122)
Supplement: Supplemental Material [file ZEPT_A_1486122_SM7585.zip › Supplementary Material A.pdf]

Interview Topic Guide_ Families/ Community Members

I will begin with asking you some questions about your experiences when you realized that water was going to come into your community and street and your home."

1. When did you realize that water might come into your house and that you might not be able to stay at your home till the water recedes?
   Probes: What do you remember from that time?
2. What did you do when you found out that you can't stay in your house?
   Probes: Where did you go?
   How did your family members feel about moving then?
   If they have children: What did you tell your children?
   How else did you prepare them?
   How did the children react?
3. Is there something else you want to tell me about your experiences before the floods really hit your community/ road/ house?

"Next, I am going to ask you a few questions about your experiences during the floods."

1. Where were you and your family during the flood?
2. What are your memories of the flood?
3. What did you think would happen to you and your family then?
4. Tell me a bit about you and your family members being able to work during the floods?
   Probes: What made it difficult to work?
   What kinds of things facilitated being able to go to work?
5. What services (through NGOs, or the government) were you able to avail during the floods?
6. Is there something else you want to tell me about your experiences during the floods?

"Now, let us talk about your experiences after the floods – this can be a time that you were sure that the rains had stopped and the cleaning started and can include experiences till recently or even those times that are affecting you currently too."

1. When did you return your house?
   Probes: What steps did you have to take in order to return to your house?
2. What kinds of difficulties did children have returning to school (e.g. not wanting to leave you to get to school, or not wanting to go by themselves, etc.)?
3. What kinds of difficulties did the children have (if any) at home after the floods (*give examples only if they cannot answer this question* - for example – maybe with playing with friends by themselves, or going out by themselves or wetting the bed, or any other health related problems like frequent stomach aches, difficulty falling asleep or nightmares, etc.)?
4. Tell me about the help did you receive after the floods? [It maybe related to child care, cleaning the house or other things like food, water, etc.]
5. How did your neighbours/ community help you?
6. How did you help you neighbours/ community?
7. What were the biggest challenges for you after the floods?
8. Tell me about some of things you or your children are proud of from that time.
9. Is there something else you want to tell me about your experiences after the floods?

"Lastly, think about your whole experience (before, during and after) the floods while you answer this question."

1. Thinking back to that time, what is the single biggest memory you have of the event and its effects on (1) you, (2) your family, and (3) your community?
2. If floods like this were to happen again, what kinds of things would you do differently?
   Probe: what instructions would you be giving/ things would you like to tell your children?
