# Supplementary material for: Children in the 2015 South Indian floods: community members’ views
Source: Eur J Psychotraumatol. 2018 Jun 26;9(Suppl 2):1486122. doi: 10.1080/20008198.2018.1486122 (PMC6038026; doi:10.1080/20008198.2018.1486122)
Supplement: Supplemental Material [file ZEPT_A_1486122_SM7585.zip › Supplementary Material B.pdf]

Interview Topic Guide_Staff of community organisations

| Name: | ID: | Age: | Gender: |
|---|---|---|---|
| Flood Severity [1- mild – 5 - severe]: | Role in the organisation: | Previous experience of such events: | Organisation: |

*"Let us begin with talking about your experiences when you realized that water was going to come into the community you were working in."*

Note: Ask for specific examples and elaboration (as necessary), especially when talking about their observation of the community/ community action.

1. What are your memories from the floods when you realized water was going to enter into the communities you lived or were working in?
2. What did the families do when they had to leave their home because of the water?
3. What strengths did you notice in the community during that time?
4. What challenges did you notice for the community during that time?
5. What else do you think was important at that time?

*"Next, I am going to ask you a few questions about your experiences during the floods."*

1. How did you (personally) help the communities you worked in during the floods?
2. How did families help each other during the floods?
3. What kinds of programs were created for the children in the community (during and after the floods)?
4. What kinds of help did your organisation offer the affected communities?
5. What kinds of help did the communities or families avail from you?
6. What else do you find important?
7. (If the staff's community/ house was flooded too) How did you manage your own situation while you were helping other communities?

*"Now, let us talk about your experiences after the floods – this can be a time that you were sure that the rains had stopped and the cleaning started and can include experiences till recently or even those times that are affecting you currently too."*

1. What do you think was the difference between those communities that recovered well versus those communities that didn't recover as well as others?
2. What kinds of programs related to the floods are currently running?
   *Probe:* What are your thoughts on current programs (since it's been about a year since the floods) helping flood affected families?
3. How else could other agencies (govt – eg. Panchayat, NGOs, school, etc.) help children and families prepare better for disasters?
4. What services you think need to be offered for these flood affected families, especially for children in these families?

*"Lastly, think about your whole experience (before, during and after) the floods while you answer this question."*

1. Thinking back to that time, what is the single biggest memory you have of the event and its effects on (1) you, and (2) the community you worked with?
2. If floods like this were to happen again, what kinds of things would you advice your organisation do differently?
3. If floods like this were to happen again, what kinds of things would you advice the community do differently?
   *Probe:* What specific steps should the community take to ensure the safety and wellbeing of the children in the community?
