# Supplementary material for: Children in the 2015 South Indian floods: community members’ views
Source: Eur J Psychotraumatol. 2018 Jun 26;9(Suppl 2):1486122. doi: 10.1080/20008198.2018.1486122 (PMC6038026; doi:10.1080/20008198.2018.1486122)
Supplement: Supplemental Material [file ZEPT_A_1486122_SM7585.zip › Supplementary Material C.pdf]

Topic Guide_FGD

Group ID:	No. of participants:	Gender (M): (F):	Organisation:	Flood Severity [1- mild – 5 - severe]:

1. What kinds of interactions did you observe/ notice families were having with their children about the flood or coping from the flood?
2. What do you think were the three most important needs of the communities you were working with?
3. What do you think were the three most important needs of the children in the communities you were working with?
4. If floods like this were to happen again, what kinds of things would you think is needed to be done differently in order for children & families in poverty to be prepared better?
5. What the three most difficulties or challenges children face in this community (beyond the financial constraints) in order to be healthy and well?
   Probe: give some specific examples
6. Resilience is defined as "the process of adapting well in the face of adversity, trauma, tragedy, threats or significant sources of stress". It's also called as "bouncing back from stress". Do you think we should work towards developing resilience in children? What do you think we need to do in order to build resilience in children – whether it is to face day-to-day life challenges, or family issues or related to disaster events like the floods?

"Next, I will read out and give you an example of the kinds of experiences I have been hearing lately. This is not any one person's experience, I have made this story up using some of the experiences I heard."

A case example: "Nalini is an 8 year old girl living with her parents, her older sister (15) and a younger brother (2 yrs). Both her parents work. On the day that water rose to a level where the water came into their house, her parents were out at work. Schools were closed and consequently, she and her siblings were home. Nalini was really worried about keeping her dolls and her school books safe, while her sister was more worried about the house computer, and her dresses amongst other things. Both children weren't sure what in the kitchen needed to be done because, the gas cylinder is too heavy and the stove is already on a height and can't get it any higher because of the length of the pipe. Most of all, they had been instructed to not touch the gas stove and the cylinder by their parents. Finally, when the water comes into the house, the siblings though were initially scared, jumped in the water – and started to play, swim, etc. Their neighbours noticed that the house had taken in water and that the kids were playing in the water. They came and asked the kids to go with them. Nalini and her brother left with the neighbours despite their protest and her sister stayed behind so that she could inform their parents and evacuate with them. Due to the urgency of the situation, Nalini and her brother did not have any clothes or essentials packed and had to leave in the clothes they were wearing. Nalini was very scared till she saw her parents and her younger brother was quite scared too and was crying. Her parents instructed her and her younger brother to continue staying with the neighbour's family. The neighbours noticed that Nalini became quiet, but, otherwise kept asking about her older sister and her parents. Nalini wasn't able to sleep well because she was scared and she found it hard to wash herself or use toilets due to lack of privacy and the relatively long distance she had to travel for them. Nalini also wouldn't let her younger brother out of sight and got nervous if he was not around for even a short period of time. The

neighbours struggled with entertaining Nalini – they were also worried water spreading into the house they were currently in. Furthermore, they were worried about their house in Nalini's neighbourhood and the condition they would find that in. They weren't sure what they would need to do in order to make it fit to live in again. Nalini's parents also had similar problems and were struggling to get information about their daughter and vice versa. The neighbours didn't know how to help Nalini or her brother calm down.

"Keeping this story in mind lets discuss somethings that might be useful to do to prepare for an event like this in the future."

1. Before starting with specific questions, what are you initial thoughts here?
2. In this situation, what are the most important things for Nalini? Her parents? Her siblings? Her community?
3. In this situation, what do you think could have helped Nalini? Her parents? her siblings? her neighbours?
4. What kinds of things would have helped Nalini and her siblings learn about floods to keep them safe?
5. How could we help Nalini clam down when she felt scared?
6. What kinds of things could Nalini's parents or school or community centre (anganwadi) do to help Nalini prepare for such events in the future?
